# A Cross-Border Biorisk Toolkit for Healthcare Professionals

**DOI:** 10.3390/ijerph21091261

**Published:** 2024-09-23

**Authors:** Pierre Vandenberghe, Jessica S. Hayes, Maire A. Connolly, Jean-Luc Gala

**Affiliations:** 1 Centre for Applied Molecular Technologies (CTMA), Institute for Clinical and Experimental Research (IREC), Université Catholique de Louvain, Tour Claude Bernard, Avenue Hippocrate, 54–55, bte B1.54.01, 1200 Bruxelles, Belgium; p.vandenberghe@uclouvain.be; 2 School of Health Sciences, College of Medicine, Nursing and Health Sciences, University of Galway, H91 TK33 Galway, Ireland; jessica.hayes@universityofgalway.ie (J.S.H.); maire.connolly@universityofgalway.ie (M.A.C.)

**Keywords:** biohazard, biothreat, biosafety, monkeypox, COVID-19, pandemic, cross-border response, caregivers, first responders, mobile laboratory, guidelines

## Abstract

The COVID-19 pandemic posed significant challenges to public health, exposing first responders to high biosafety risks during medical assistance and containment efforts. The PANDEM-2 study aimed to address these critical biosafety issues by emphasising the importance of frequently updated, harmonised guidelines. This study reviewed scientific publications, lessons learned, and real-world experiences from the COVID-19 pandemic to identify biorisk gaps in three critical areas: (i) patient transportation and management, (ii) sample handling and testing, and (iii) data management and communication by laboratory staff. At the onset of the pandemic, first responders faced several challenges, including the rapid expansion of emergency medical services, conversion of non-medical structures, increased internal and cross-border transport of infected patients, frequent changes in biosafety protocols, and a shortage of personal protective equipment. In response, this study developed a versatile and easily adaptable toolkit, including biosafety guidance and recommendations linked to updated national and international online repositories. It establishes the groundwork for a minimum standard that can be tailored to various pandemic response scenarios, using monkeypox as a fictive test case. The toolkit enables rapid access to updated information via QR codes and mobile devices, improving biorisk response by providing an adaptable and standardised approach for caregivers involved in national and cross-border responses.

## 1. Introduction

The COVID-19 pandemic, caused by the novel severe acute respiratory syndrome coronavirus 2 (SARS-CoV-2), has presented unprecedented challenges to public health systems worldwide [1]. During this global health crisis, first responder practitioners from police, civil protection, firefighters, and emergency medical teams have been at the forefront of providing medical assistance and containment efforts, which has exposed them to significant biosafety risks. The highly infectious nature of SARS-CoV-2 requires strict adherence to biosafety protocols to protect both the first responders and the communities they serve. Failure to take appropriate precautions could lead to further transmission of the virus, potentially exacerbating the pandemic’s impact and straining healthcare systems [2,3]. To reduce the workload on overburdened hospitals, field hospitals and mobile laboratories were deployed both nationally [4,5] and internationally [6], to address the patient overflow and testing needs. Furthermore, hospitals in less severely affected countries provided assistance by accepting patients from overburdened healthcare systems in other countries [7]. The need for such a cross-border response within Europe has further complicated the situation by adding complexities related to international collaboration and coordination, the harmonisation of safety protocols, and potential exposure to multiple virus variants. Differences in testing capacities, personal protective equipment (PPE) availability, and vaccination rates may create discrepancies in protection levels for first responders operating across borders [8]. Thus, harmonising and improving biosafety guidelines and protocols is of utmost importance and requires a delicate balance between respecting each country’s autonomy in managing the pandemic, the necessity for collective global action, and listening to the voice of the first responders.

In this context, the Horizon 2020-funded European project PANDEM-2 (Pandemic Preparedness and Response) sought to develop new solutions for effective pandemic management across the EU. In a previous study that was part of this project, we surveyed the biosafety challenges faced by first responders at the National Institute of Medical Emergency (INEM) in Portugal. The INEM was taken as a representative operational model for the national first responder agencies of the European member states because of their critical role during the COVID-19 pandemic. The previous survey asked for questions and opinions on current biosafety protocols, technical concerns during patient transport, and issues after the patients arrived at the hospital. Key findings revealed concerns about risk assessment, the inadequacy of guidelines, and disparities in equipment access [9]. This recent survey laid the foundation for promoting EU cross-border response to health emergencies through advances in information technology solutions and pandemic preparedness training. In the current follow-up study, we present concrete solutions developed for improving biosafety in a global pandemic context, based on an extensive review of the existing literature, lessons learned, and analysis of real-world experiences from first responders. 

## 2. Materials and Methods

### 2.1. Biosafety Challenges and Solutions for Stakeholders: Insights from the PANDEM-2 Project

This study is part of the project PANDEM-2 (Pandemic Preparedness and Response) (Website: https://pandem-2.eu/ (accessed on 18 September 2024); LinkedIn: https://www.linkedin.com/company/pandem-2/ (accessed on 18 September 2024); Twitter:@PANDEM2H2020). This project responded to the H2020-SU-SEC-2018-2019-2020 call on “Demonstration of novel concepts for the management of pandemic crises”. The members of the consortium included EU experts in a variety of fields, including health, security, defence, microbiology, communications, information technology, and crisis management. Target end-users are stakeholders who may be exposed to biorisk during their on-site intervention, as well as those managing the crisis from a distance or involved in making directives and guidelines. This includes first responders, second responders, and policymakers.

### 2.2. Biosafety Documentation Research Strategy

As biosafety exists at all levels of healthcare, this study focused on strategic issues with a significant impact on the pandemic situation and evolution. In collaboration with biosafety experts and first responders from the consortium, the three following key topics were defined: (1) patient transportation and management, (2) handling and testing of samples, and (3) data management and communication among stakeholders of mobile laboratories and newly established medical structures. 

A three-step search for biosafety documentation relevant to those topics was conducted. The first step involved conducting an exhaustive search and collection of existing biosafety guidelines using “Biosafety” and “Biosecurity” keywords from international organisations such as the World Health Organisation (WHO), the European Centre for Disease Prevention and Control (ECDC), and the Centers for Disease Control and Prevention (CDC), which served as foundational references. The second step involved obtaining biosafety protocols from consortium partners, including those from the Red Cross first responders, a university medical centre, and a WHO’s Global Outbreak Alert and Response Network (GOARN)-affiliated mobile laboratory. Additionally, an internet search was carried out to identify relevant best practices used by first responders globally, through Google Search and PubMed. The following keywords were used to retrieve relevant documents: “SOP patient transport”, “COVID-19 patient transport”, “Patient transport HID”, “Biosafety SOP”, “COVID-19 biosafety SOP”, “Pathogen inactivation”, “SARS-CoV-2 inactivation”. A survey was also submitted to obtain first responders’ opinions about biosafety guidelines [9]. The final step involved a comprehensive review of lessons learned from the COVID-19 pandemic, particularly focusing on biosafety aspects. Reports from other European projects of the Preparedness and Response for Emergency Situations in Europe (PREPARE) cluster and research papers documenting successful practices and challenges encountered by first responders were analysed and used as a basis for identifying gaps and opportunities for improvement in biosafety measures. The database of the retrieved biorisk documents is available in Supplementary Materials S6.

### 2.3. Projection of a Fictive Scenario Using Monkeypox as a Test Case

While our toolkit was primarily designed to respond to a new pandemic, it also aimed to improve the preparedness of first responders for future pandemics. Although initially developed during the COVID-19 pandemic with a focus on the SARS-CoV-2 virus, it is intended to be applicable to other pandemic agents. We chose monkeypox as a test case to assess the adaptability and robustness of IT solutions and the toolkit for first responders, as this new outbreak began in several European countries in May 2022, coinciding with the duration of this study.

## 3. Results

### 3.1. Identification of Biosafety Gaps and Concerns

We identified five common issues from the lesson learned documentation (points 1 to 5) and three major issues faced by first responders based on the survey of our previous study (points 6 to 8) [9]:

1. The need to quickly expand the workforce capacities of emergency medical systems (EMSs) led to the recruitment of volunteers or junior doctors who lacked biosafety training or experience.
2. Due to overcrowding in intensive care units (ICUs), some hospitals were forced to transfer COVID-19-positive patients to other hospitals and, in some cases, to other countries. This resulted in a rise in demand for modes of transportation, some of which, such as air transfers by helicopter or airplane, lacked clear biosafety guidelines.
3. Non-medical structures were rapidly converted into field hospitals to admit COVID-19 patients in countries where hospital capacity was exceeded. This led to issues with biosafety protocols in some situations where the infrastructure provided was not equivalent to healthcare facilities (for example, lack of a waste management system).
4. A shortage of personal protective equipment at the outset of the pandemic and the discomfort of wearing them all day raised safety concerns among first responders.
5. Increased demand for SARS-CoV-2 testing placed additional constraints on clinical and laboratory personnel who perform sampling and diagnosis, potentially increasing the risk of biosafety failure.
6. During the COVID-19 pandemic, biosafety protocols were frequently changed, making them difficult to follow.
7. An overabundance of caution and a lack of risk assessment were also noted.
8. Some protocols have not been adapted to the realities of “street emergencies”, where the environment is chaotic, high-risk, and lacks the level of control found in laboratories or hospitals.

These gaps underline the importance of just-in-training and quick availability of updated biosafety recommendations among first responders.

### 3.2. Guidance for First Responders, Clinicians, and Laboratory Personnel

**Figure 1:**
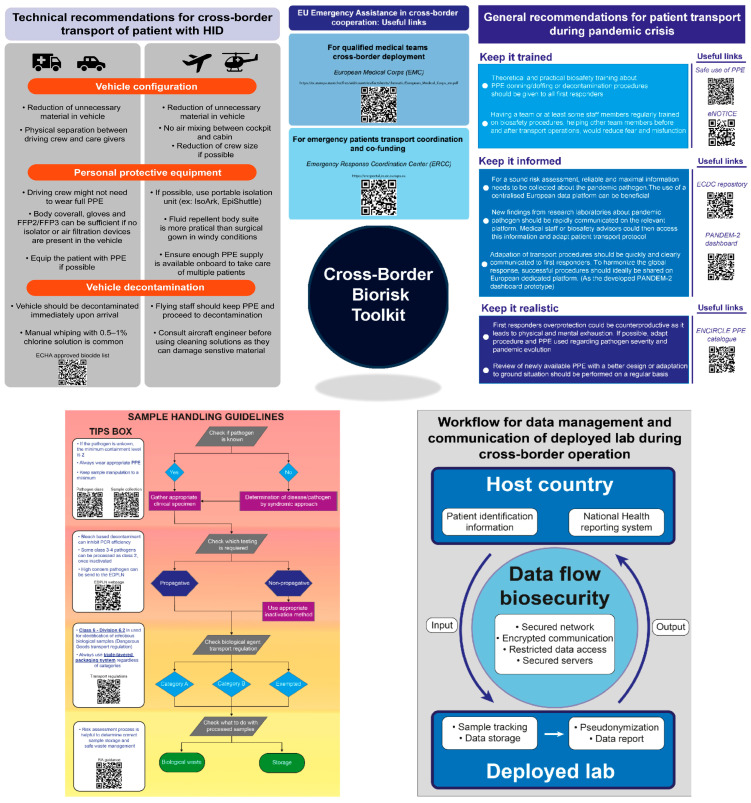
Overview of the cross-border biorisk toolkit.

Based on the identified gaps, we developed a toolkit containing guidance and recommendations for each of the three defined biosafety topics. The first set of guidelines addressed (i) the transportation and management of patients with highly infectious diseases (HID). Two useful links are available to help stakeholders find EU assistance for cross-border responses through the Emergency Response Coordination Centre and the European Medical Corps, which provide logistical, medical, and diagnostic support. Technical recommendations were also developed to guide first responders in choosing vehicle and staff configuration during cross-border transport, ensuring compliance with minimal biosafety requirements. Finally, general recommendations for patient transport in a pandemic context were generated together with QR codes, leading to relevant resources that will help stakeholders set up those recommendations (Figure 1). The second set of guidelines provided are related to (ii) the sample handling and testing. A decision tree with tip boxes was designed to help stakeholders who have different scientific backgrounds to quickly, correctly, and safely process a sample during an outbreak (Figure 1). More detailed information supporting this decision tree can be found in the publicly available PANDEM-2 repository [10].

The final set of recommendations focused on (iii) data management and communication among stakeholders working in field hospitals and mobile laboratories. A general workflow was established to explain which party needs to provide which information and how sensitive data must be processed and protected during the operation (Figure 1). Again, more detailed information can be obtained from the publicly available PANDEM-2 repository [10]. Supplementary Materials Figures S1–S5 provide full high-resolution versions of the QR codes and printable versions of the flyers.

### 3.3. Test Case: Monkeypox

A guideline toolkit, developed and applied to the context of the recent monkeypox pandemic, focused on establishing protocols for the transport of suspected monkeypox patients, recognising monkeypox infection, identifying the causative pathogen, and safely handling patient samples.

#### 3.3.1. Transport of Suspected Monkeypox Patients

Understanding the clinical features of monkeypox is critical for effective risk assessment and preparing EMS personnel. The “Keep it Informed” (Supplementary Materials Figure S2) flyer links to the ECDC’s disease repository alphabetically. By selecting “monkeypox”, you are directed to a “Fact sheet” with pertinent information about the disease. This sheet provides the following information:

- Monkeypox is resistant to drying and can survive on surfaces for extended periods of time.
- It is susceptible to common disinfectants.
- Transmission takes place through respiratory droplets during close or prolonged face-to-face interactions, direct contact with an infected person’s body fluids, or touching contaminated surfaces.
- UPDATE: Sexual transmission has now been determined to be a major factor in the current outbreak.

According to these results, ambulance personnel can use appropriate PPE to prevent direct contact with the patient’s body fluids and droplets (refer to the ECDC’s PPE use guidance in the “Keep it Informed” flyer for more information). The recommended PPE includes gloves, coats, gowns, footwear covers, and surgical masks. To minimise droplet transmission, patients should wear a surgical mask if possible (Supplementary Materials Figure S2). Considering monkeypox’s ability to survive on surfaces for extended periods, ambulance personnel should limit the items they bring into the vehicle to those that are absolutely necessary, reducing the risk of contamination. Upon the patient’s arrival at the hospital, the ambulance must be promptly decontaminated, with crews donning PPE. Since monkeypox is susceptible to common disinfectants, a chlorine solution is suitable for cleaning the equipment. Furthermore, used bedding should be securely packed into biohazard-labelled containers to prevent accidental contamination by hospital cleaning staff as outlined in the “Technical recommendations” flyer (Supplementary Materials Figure S3).

#### 3.3.2. Recognising Monkeypox Infection, Identifying the Causative Pathogen, and Handling Samples

If a healthcare professional is faced with a differential diagnosis that includes monkeypox and needs to confirm the diagnosis but is unfamiliar with the specific procedures, the flowchart “Specimen handling guidelines” may be useful. The confirmation of the diagnosis requires the identification of the pathogen. If medical personnel are unsure which class of biological agent the virus belongs to, they can identify it by scanning the QR code labelled “Class of Pathogen” printed in the flowchart’s advice box, as shown in Supplementary Materials Figure S4. 

Healthcare professionals must wear the appropriate PPE (gloves, eye protection, gown, and respiratory protection) when collecting clinical specimens that are likely to contain a class 3 pathogen. In Supplementary Materials Figure S4, readers will find a QR code that links to the WHO’s recommended list of clinical samples for pathogen identification testing. In the context of our study, monkeypox is described as a “dermatological syndrome”, which requires the collection of scabs or swabs from lesions. The qPCR analysis, often necessary to confirm the identification of an infectious agent, is classified as a non-propagative analysis. As such, the sample can be carried out in a tube containing the Inactivation Transport Medium (ITM), which includes guanidine salts and ionic detergents that lyse viruses and cells to release proteins and nucleic acids (Supplementary Materials Figure S4). Samples can be sent to a reference laboratory if the facility lacks the necessary expertise or reagents. Although the sample is already inactivated in the ITM tube, a three-layer packaging system remains mandatory. However, no specific size recommendations are required for air, sea, or ground shipping. The outer layer must be labelled and indicate the content. If the sample is no longer needed and testing capabilities are available in-house, it can be safely disposed of in leak-proof containers, such as a biological waste bin, following appropriate risk assessment for biological waste management. If additional testing is required, it should be stored in a biosafety room.

## 4. Discussion

Based on a thorough review of guidelines and lessons learned from the COVID-19 pandemic, this study identified significant gaps and needs in biorisk management across Europe, whether for natural, accidental, or deliberate releases of high-consequence pathogens. We selected three key biorisk topics based on the first-hand experiences of toolkit end-users. While basic resources such as WHO monographs [11,12,13,14] and CDC Biosafety and Tools resources [15] have assisted first responders during crises, their static nature has limitations. A wealth of information on various biosafety topics is available in separate documents or weblinks, requiring users to spend time and effort to find relevant information. Additionally, printed guidelines lack real-time update capabilities, which are critical in rapidly evolving pandemic scenarios with emerging biological agents, particularly those with gain-of-function modifications resulting from synthetic biology or gene editing [16]. Relying on outdated written information may lead to obsolete practices, increasing the risk of biosafety and biosecurity breaches [17]. Therefore, integrating traditional resources with advanced communication technologies [18] and information sharing is essential [19,20]. A system comparable to the current toolkit could convert these resources into living documents that respond in real time to global health threats.

The collapse of several European healthcare systems highlighted the importance of efficient patient transfer, medical assistance, and increased testing capacities. To address these major issues, rapid changes were implemented, including an increased volunteer pool [21], alternative patient transportation methods [22,23], the conversion of public buildings into temporary hospitals [24,25], and the establishment of testing centres [26]. However, these solutions were not always implemented in accordance with updated biorisk guidelines. Our findings underscore the importance of international collaboration in constantly revising emergency guidelines to manage emerging health crises involving high-consequence pathogens.

Given the importance of swift, reliable responses in health crisis management [27], we adopted a flyer format with integrated QR codes to provide visually attractive and easily accessible information directly linked to online reference repositories, like WHO and the ECDC. Our biorisk toolkit offers a dynamic solution for timely communication in healthcare settings. Given the smartphone availability at work, QR codes provide an efficient means of data transmission, ensuring immediate updates and access to the most recent guidelines [28,29]. As highlighted in the fictive scenario with monkeypox, this feature is especially critical during pandemics caused by unexpected or new emerging agents when protocols must evolve quickly, as in the case of COVID-19.

Our biorisk toolkit is designed to be understandable to a broad audience, thereby maximising its impact. It provides up-to-date content while ensuring that first responders across borders have access to consistent and current information. To address a variety of response scenarios, recommendations are tailored as “minimum standards,” taking into account healthcare infrastructure, available resources, and the challenges that frontline workers face throughout Europe. This approach ensures alignment and appropriate response in terms of personal protection, risk assessment, patient management, decontamination, reporting, and post-exposure surveillance [30]. Our layered approach provides generic information to first responders (police officers, firefighters, civil protection teams, paramedics), detailed information to specialised responders (HAZMAT teams, laboratory staff, and medical professionals), and comprehensive data to advanced response teams. This toolkit serves as a bridge between different users, allowing them to better understand their roles and collaborate more effectively.

The WHO’s GOARN recently emphasised the importance of collaboration, coordination, and communication between first responders and laboratory staff, as well as biosafety and biosecurity, through a series of simulation field exercises in which mobile laboratories and emergency medical services (EMSs) dealt with a fictitious avian influenza pandemic affecting populations [31]. These organisations had established well-defined biorisk standard operating protocols specific to their respective fields of work, such as personal protection and waste management [32] or data communication between stakeholders [33]. However, there was a clear lack of harmonisation in procedures to ensure interoperability among health professionals. This lack of coordination reduced the effectiveness of collaborative efforts between medical and laboratory staff, as both groups had to spend more time understanding each other’s protocols and procedures. This inefficiency could cause response time delays and potential miscommunications, ultimately impacting the effectiveness of biorisk management and overall safety. This underlies the importance of developing a toolkit providing easily accessible, relevant, and harmonised guidelines such as the one presented in this study. While intended for a wide range of stakeholders, the toolkit has limitations. English-language flyers may challenge non-English speakers, as observed with our consortium end-users from various European countries. We are considering translating the toolkit into other key languages to enhance accessibility across Europe. Another anticipated limitation is ensuring that the links associated with QR codes remain functional over time. Documents hosted online may indeed be removed, modified, or relocated, rendering QR codes obsolete and requiring manual updates, which can be time-consuming. Artificial intelligence could provide a solution through regular web scraping and automatic QR code updates via specifically designed algorithms. This AI-based automation would also help account for cultural differences between EU member states and regional practices. To identify areas for improvement, first responders are currently evaluating the toolkit as part of the European project eNOTICE-2 (EU Network of Training Centres for Preparedness to CBRN Events, UCPM-2023-KAPP-PREP, https://civil-protection-knowledge-network.europa.eu/projects/enotice-2 (accessed on 18 September 2024)).

## 5. Conclusions

This study identified significant gaps in biorisk management across Europe during the COVID-19 pandemic. The urgent need for EMS expansion, ICU overcrowding, the conversion of non-medical structures into field hospitals, PPE shortages, and SARS-CoV-2 testing surges highlighted the challenges of adapting and adhering to biosafety guidelines across borders. In response, we developed a toolkit that provides rapid and successive information updates during crises and makes recommendations to stakeholders on biorisk issues related to patient transportation, sample and data management, and the development of “EU-friendly” guidelines.

## Data Availability

The data presented in this study are available upon request from the corresponding author.

## Supplementary Materials

The following supporting information can be downloaded at: https://www.mdpi.com/article/10.3390/ijerph21091261/s1, Figure S1: EU emergency assistance in cross-border cooperation; Figure S2: Technical recommendations for cross-border transport of patient with HID; Figure S3: General recommendations for patient transport during pandemic crisis; Figure S4: Sample handling guidelines; Figure S5: Workflow for data management and communication of deployed lab during cross-border operation. Supplementary Material S6: Database of biorisk documents retrieved from international organization websites, Mendeley and Google Search.
