# Supplementary material for: A Cross-Border Biorisk Toolkit for Healthcare Professionals
Source: Int J Environ Res Public Health. 2024 Sep 23;21(9):1261. doi: 10.3390/ijerph21091261 (PMC11431820; doi:10.3390/ijerph21091261)
Supplement: Supplementary file 1 [file ijerph-21-01261-s001.zip › Supplementary material S6_Document database.pdf]

Database of biorisk documents retrieved from international organization websites, Mendeley and Google Search

Keywords:

"Biosafety";" Biosecurity"; "SOP patient transport";" COVID-19 patient transport"; "Patient transport HID"; "Biosafety SOP"; "COVID-19 biosafety SOP"; "Pathogen inactivation"; "SARS-CoV2 inactivation"

[1] World Health Organisation, "Laboratory Biosafety Manual Monograph Risk Assessment", 2020. https://www.who.int/publications/i/item/9789240011458

[2] World Health Organisation, "Guidance on implementing regulatory requirements for biosafety and biosecurity in biomedical laboratories-a stepwise approach", 2020. https://apps.who.int/iris/bitstream/handle/10665/332244/9789241516266-eng.pdf?sequence=1&isAllowed=y

[3] World Health Organisation, "Laboratory biosafety manual fourth edition and associated monographs personal protective equipment", 2020. https://www.who.int/publications/i/item/9789240011410

[4] World Health Organisation, "MODULE 17: Management of Specific Infectious Wastes." https://cdn.who.int/media/docs/default-source/wash-documents/wash-in-hcf/training-modules-in-health-care-waste-management/module-17---management-of-specific-infectious-waste.pdf?sfvrsn=8a2e246d_2

[5] CDC, "Best Practices for Environmental Cleaning in Healthcare Facilities: in Resource-Limited Settings Version 2." https://www.cdc.gov/hai/pdfs/resource-limited/environmental-cleaning-RLS-H.pdf

[6] CDC, "Biosafety in Microbiological and Biomedical Laboratories 6th Edition Centers for Disease Control and Prevention National Institutes of Health", 2020. https://www.cdc.gov/labs/pdf/SF__19_308133-A_BMBL6_00-BOOK-WEB-final-3.pdf

[7] M. L. Ruiu, "Mismanagement of Covid-19: lessons learned from Italy," *Journal of Risk Research*, vol. 23, no. 7–8, 2020, doi: 10.1080/13669877.2020.1758755.

[8] C. Coughlan *et al.*, "COVID-19: Lessons for junior doctors redeployed to critical care," *Postgraduate Medical Journal*, vol. 97, no. 1145, 2021, doi: 10.1136/postgradmedj-2020-138100.

[9] F. C. Fang *et al.*, "COVID-19-Lessons Learned and Questions Remaining," *Clinical Infectious Diseases*, vol. 72, no. 12, 2021, doi: 10.1093/cid/ciaa1654.

[10] A. Barkia, H. Laamrani, A. Belalia, A. Benmamoun, and Y. Khader, "Morocco's National Response to the COVID-19 Pandemic: Public Health Challenges and Lessons Learned," *JMIR Public Health and Surveillance*, vol. 7, no. 9, p. e31930, Sep. 2021, doi: 10.2196/31930.

[11] K. Wang, X. Zhu, and J. Xu, "Laboratory Biosafety Considerations of SARS-CoV-2 at Biosafety Level 2," *Health Security*, vol. 18, no. 3. Mary Ann Liebert Inc., pp. 232–236, May 01, 2020. doi: 10.1089/hs.2020.0021.

[12] A. Zangrillo and L. Gattinoni, "Learning from mistakes during the pandemic: the Lombardy lesson," *Intensive Care Medicine*, vol. 46, no. 8. Springer, pp. 1622–1623, Aug. 01, 2020. doi: 10.1007/s00134-020-06137-9.

[13] K. K. W. To *et al.*, "Lessons learned 1 year after SARS-CoV-2 emergence leading to COVID-19 pandemic," *Emerging Microbes and Infections*, vol. 10, no. 1. Taylor and Francis Ltd., pp. 507–535, 2021. doi: 10.1080/22221751.2021.1898291.

[14] NO FEAR, "Network Of practitioners For Emergency medicAl systems and cRitical care," 2020.

[15] NO FEAR, "Summary of main findings, gaps and lessons learned from M24 to M30 Network Of practitioners For Emergency medicAl systems and cRitical care," 2019.

[16] F. Pfäfflin *et al.*, "Preparing for patients with high-consequence infectious diseases: Example of a high-level isolation unit," *PLoS ONE*, vol. 17, no. 3 March, pp. 1–16, 2022, doi: 10.1371/journal.pone.0264644.

[17] A. Baka *et al.*, "A curriculum for training healthcare workers in the management of highly infectious diseases.," *European communicable disease bulletin*, vol. 12, no. 6, pp. 1–5, 2007, doi: 10.2807/esm.12.06.00716-en.

[18] SOP-Liberia, "Ebola virus disease (EVD) patient transport". https://pdf.usaid.gov/pdf_docs/PA00KBG1.pdf

[19] SOP-Ministry of Health and Family Welfare Directorate General of Health Services "Transporting a suspected/confirmed case of COVID-19". https://www.mohfw.gov.in/pdf/StandardOperatingProcedureSOPfortransportingasuspectorconfirmedcaseofCOVID19.pdf

[20] CDC, "Example: Standard Operating Procedure (SOP) for Patient Handoff between a Healthcare Facility and a Transporting Ambulance." https://www.cdc.gov/vhf/ebola/pdf/patient-handoff.pdf

[21] P. P. Bredmose *et al.*, "Decision support tool and suggestions for the development of guidelines for the helicopter transport of patients with COVID-19," *Scandinavian Journal of Trauma, Resuscitation and Emergency Medicine*, vol. 28, p. 43, 2020, doi: 10.1186/s13049-020-00736-7.

[22] E. J. Spoelder, M. C. T. Tacken, G.-J. van Geffen, and C. Slagt, "Helicopter transport of critical care COVID-19 patients in the Netherlands: protection against COVID-19 exposure-a challenge to critical care retrieval personnel in a novel operation", doi: 10.1186/s13049-021-00845-x.

[23] R. Albrecht, J. Knapp, L. Theiler, M. Eder, and U. Pietsch, "Transport of COVID-19 and other highly contagious patients by helicopter and fixed-wing air ambulance: A narrative review and experience of the Swiss air rescue Rega," *Scandinavian Journal of Trauma, Resuscitation and Emergency Medicine*, vol. 28, no. 1. BioMed Central Ltd., May 14, 2020. doi: 10.1186/s13049-020-00734-9.

[24] SOP-NIU Mater Misericordiae University Hospital, "Transport of Infected Patients Contents". https://www.hpsc.ie/a-z/vectorborne/viralhaemorrhagicfever/guidance/vhfguidancechapters/Chp%206_Transport_final1.pdf

[25] S. Schilling *et al.*, "Transportation capacity for patients with highly infectious diseases in Europe: a survey in 16 nations," *Clinical Microbiology and Infection*, vol. 21. Elsevier B.V., pp. e1–e5, Apr. 01, 2019. doi: 10.1111/1469-0691.12290.

[26] M. F. Liew, W. T. Siow, Y. W. Yau, and K. C. See, "Safe patient transport for COVID-19," *Critical Care*, vol. 24, no. 1. BioMed Central Ltd., Mar. 18, 2020. doi: 10.1186/s13054-020-2828-4.

[27] B. Bannister, V. Puro, F. M. Fusco, J. Heptonstall, and G. Ippolito, "Framework for the design and operation of high-level isolation units: consensus of the European Network of Infectious Diseases," *The Lancet Infectious Diseases*, vol. 9, no. 1. pp. 45–56, Jan. 2009. doi: 10.1016/S1473-3099(08)70304-9.

[28] World Health Organisation, "Assessment tool for laboratories implementing SARS-CoV-2 testing Interim guidance 2 October 2020 Background," 2020. https://apps.who.int/iris/bitstream/handle/10665/335843/WHO-2019-nCoV-Lab_Assessment_Tool-2020.2-eng.pdf?sequence=1&isAllowed=y

[29] CDC, "Supplement F: Laboratory Guidance - Appendix F5 - Laboratory Biosafety Guidelines for Handling and Processing Specimens Associated with SARS-CoV." https://www.cdc.gov/sars/guidance/f-lab/downloads/f-lab-full.pdf

[30] M. R. Capoor and A. Parida, "Current perspectives of biomedical waste management in context of COVID-19"," *Indian J Med Microbiol*, vol. 39, no. 2, pp. 171–178, Apr. 2021, doi: 10.1016/j.ijmmb.2021.03.003.

[31] B. Wight and M. Boss, *Decontamination and Assessment*. 2002. doi: 10.1201/9781420032161.ch10.

[32] J. Cui, F. Li, and Z. L. Shi, "Origin and evolution of pathogenic coronaviruses," *Nature Reviews Microbiology*, vol. 17, no. 3. pp. 181–192, Mar. 01, 2019. doi: 10.1038/s41579-018-0118-9.

[33] B. Hu, H. Guo, P. Zhou, and Z. L. Shi, "Characteristics of SARS-CoV-2 and COVID-19," *Nature Reviews Microbiology*, vol. 19, no. 3. pp. 141–154, Mar. 01, 2021. doi: 10.1038/s41579-020-00459-7.

[34] L. R. Petersen, D. J. Jamieson, A. M. Powers, and M. A. Honein, "Zika Virus," *New England Journal of Medicine*, vol. 374, no. 16, pp. 1552–1563, Apr. 2016, doi: 10.1056/NEJMra1602113.

[35] K. K. To *et al.*, "Additional molecular testing of saliva specimens improves the detection of respiratory viruses," *Emerging Microbes and Infections*, vol. 6, no. 6, Jun. 2017, doi: 10.1038/emi.2017.35.

[36] S. Banik *et al.*, "Inactivation of SARS-CoV-2 virus in saliva using a guanidium based transport medium suitable for RT-PCR diagnostic assays," *PLoS ONE*, vol. 16, no. 6 June 2021, pp. 1–10, 2021, doi: 10.1371/journal.pone.0252687.

[37] T. Kang, J. Lu, T. Yu, Y. Long, and G. Liu, "Advances in nucleic acid amplification techniques (NAATs): COVID-19 point-of-care diagnostics as an example," *Biosensors and Bioelectronics*, vol. 206, p. 114109, 2022, doi: https://doi.org/10.1016/j.bios.2022.114109.

[38] M. N. Anahtar *et al.*, "Clinical Assessment and Validation of a Rapid and Sensitive SARS-CoV-2 Test Using Reverse Transcription Loop-Mediated Isothermal Amplification without the Need for RNA Extraction," *Open Forum Infectious Diseases*, vol. 8, no. 2, 2021, doi: 10.1093/ofid/ofaa631.

[39] F. Gong, H. X. Wei, Q. Li, L. Liu, and B. Li, "Evaluation and Comparison of Serological Methods for COVID-19 Diagnosis," *Frontiers in Molecular Biosciences*, vol. 8. Frontiers Media S.A., Jul. 23, 2021. doi: 10.3389/fmolb.2021.682405.

[40] Alhajj M and Farhana A, "Enzyme Linked Immunosorbent Assay," StatPearls., StatPearls Publishing , 2022. Accessed: Jul. 01, 2022.

[41] C. Quince, A. W. Walker, J. T. Simpson, N. J. Loman, and N. Segata, "Shotgun metagenomics, from sampling to analysis," *Nature Biotechnology*, vol. 35, no. 9, pp. 833–844, 2017, doi: 10.1038/nbt.3935.

[42] M. W. Rosenstierne *et al.*, "Rapid bedside inactivation of Ebola virus for safe nucleic acid tests," *Journal of Clinical Microbiology*, vol. 54, no. 10, pp. 2521–2529, Oct. 2016, doi: 10.1128/JCM.00346-16.

[43] J. L. Sagripanti, B. Hülseweh, G. Grote, L. Voß, K. Böhling, and H. J. Marschall, "Microbial inactivation for safe and rapid diagnostics of infectious samples," *Applied and Environmental Microbiology*, vol. 77, no. 20, pp. 7289–7295, Oct. 2011, doi: 10.1128/AEM.05553-11.

[44] D. van Bockel *et al.*, "Evaluation of commercially available viral transport medium (VTM) for SARS-CoV-2 inactivation and use in point-of-care (POC) testing," *Viruses*, vol. 12, no. 11, Oct. 2020, doi: 10.3390/v12111208.

[45] O. Erster *et al.*, "Improved sensitivity, safety, and rapidity of COVID-19 tests by replacing viral storage solution with lysis buffer," *PLoS ONE*, vol. 16, no. 3 March, Mar. 2021, doi: 10.1371/journal.pone.0249149.

[46] C. Clarke *et al.*, "Novel molecular transport medium used in combination with Xpert MTB/RIF ultra provides rapid detection of Mycobacterium bovis in African buffaloes," *Scientific Reports*, vol. 11, no. 1, Dec. 2021, doi: 10.1038/s41598-021-86682-5.

[47] R. E. Thom *et al.*, "Evaluation of the SARS-CoV-2 Inactivation Efficacy Associated With Buffers From Three Kits Used on High-Throughput RNA Extraction Platforms," *Frontiers in Cellular and Infection Microbiology*, vol. 11, Sep. 2021, doi: 10.3389/fcimb.2021.716436.

[48] S. R. Welch *et al.*, "Analysis of Inactivation of SARS-CoV-2 by Specimen Transport Media, Nucleic Acid Extraction Reagents, Detergents, and Fixatives," 2020.
