# Supplementary figures and images for: A Cross-Border Biorisk Toolkit for Healthcare Professionals
Source: Int J Environ Res Public Health. 2024 Sep 23;21(9):1261. doi: 10.3390/ijerph21091261 (PMC11431820; doi:10.3390/ijerph21091261)

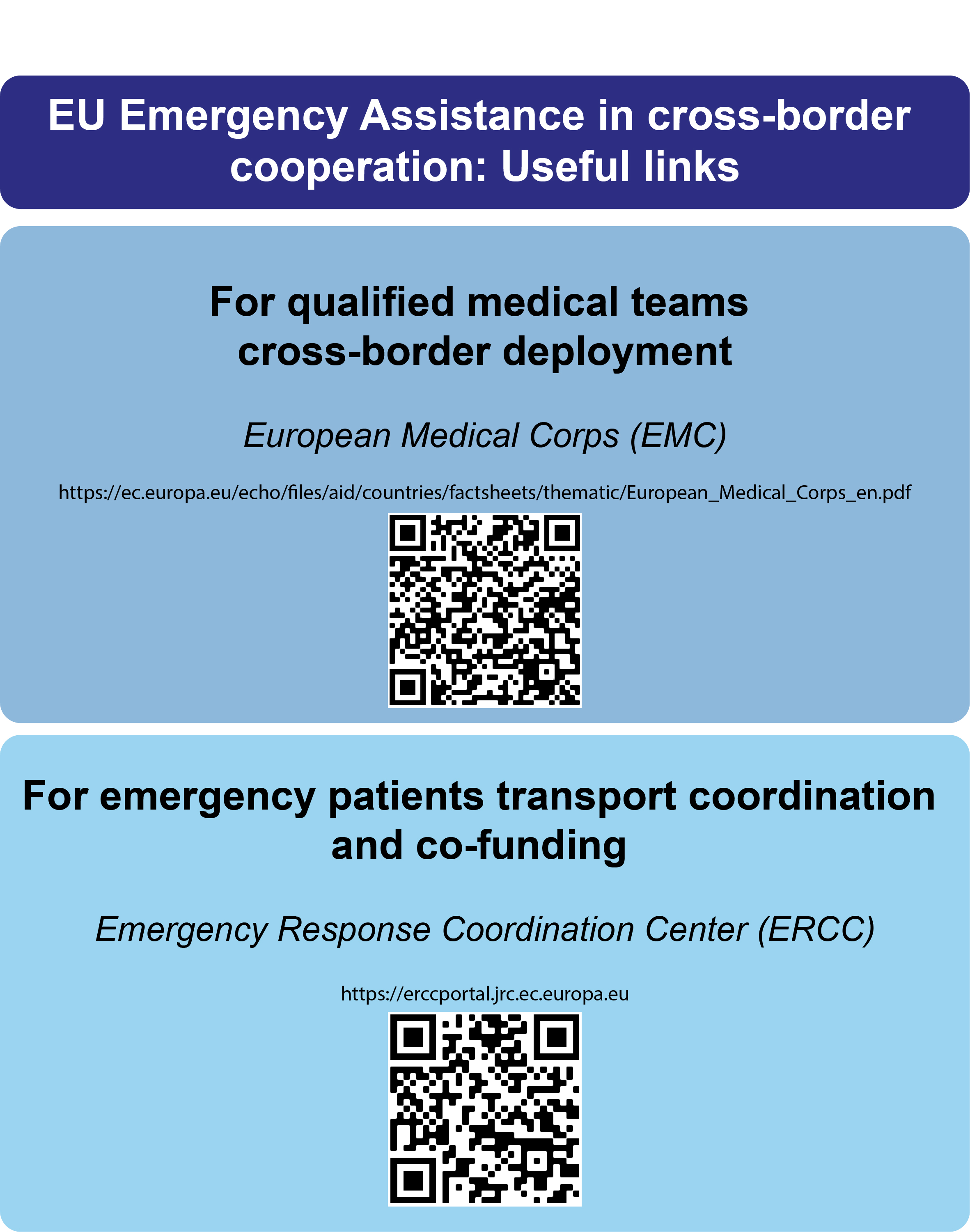

Supplement: Supplementary file 1 [file ijerph-21-01261-s001.zip › Fig S1_EU Emergency Assistance in cross-border cooperation.tif]
